# Risk of Short-Term Mortality after Intracerebral Haemorrhage due to Weekend Hospital Admission in Poland

**DOI:** 10.1155/2020/2198384

**Published:** 2020-12-09

**Authors:** Marta Nowakowska-Kotas, Marta Waliszewska-Prosół, Paulina Papier, Sławomir Budrewicz, Tomasz Bańkowski, Anna Pokryszko-Dragan

**Affiliations:** ^1^Departments of Neurology, Wroclaw Medical University, Wroclaw, Poland; ^2^Department of Cardiology, Lower Silesian Specialist Hospital, Wroclaw, Poland

## Abstract

**Background:**

The mortality rate for spontaneous intracerebral haemorrhage (ICH) has remained high and stable for many years. The unfavourable prognostic factors include age, bleeding volume, location of the haematoma, high blood pressure, and disturbed consciousness on admission. Other risk factors associated with medical care also deserve attention. The study aimed to analyse the relationship between day of admission, concerning other prognostic factors, and short-term mortality in ICH, in a Polish specialist stroke unit.

**Methods:**

Medical records of 156 patients (74 males, 82 females, mean age 68.7 years) diagnosed with spontaneous ICH and admitted to a specialist stroke center were retrospectively analysed. Demographics, location, volume of bleeding, blood pressure values, and the Glasgow Coma Scale (GCS), as well as the day of admission, were determined. The relationships were analysed between these factors and 30-day mortality in the patients with ICH.

**Results:**

A total of 83 patients were admitted to the hospital during weekdays (Monday 8 am to Friday 3 pm) and 73 during weekends or holidays. Of these, 65 patients died within 30 days. Patients admitted at weekends initially presented with lower GCS scores. Admission on Saturday was associated with an increased risk of death (OR 3.38, 95% CI 1.2–9.48, *p* < 0.05), but after correction for clinical state measured with the GCS and ICH score, the association was no longer significant.

**Conclusions:**

The time and mode of admission were not associated with increased risk of short-term mortality in ICH patients. Prehospital care issues should be additionally considered as prognostic factors of the outcome.

## 1. Introduction

Intracerebral haemorrhage (ICH) occurs with an incidence of 41–47 per 100,000 adult population of Poland, which means that it makes up almost 13–17% of stroke cases registered in the country. Unfortunately, the mortality rate remains at the stable and high level of 31.4% [1, 2]. Worldwide, it also has the highest rate of mortality among other types of strokes, and there has not been much progress in recent years in lowering the mortality rate [3–5]. Death in ICH occurs either due to irreversible brain damage and consecutive withdrawal of life-support or other medical conditions, which usually develop as complications (pulmonary embolism, infections) [6]. The early mortality rate (in 30 days) depends mainly on age and the volume of haemorrhage [7, 8] and its location, [9]especially interventricular bleeding [10], Glasgow Coma Scale (GCS), and blood pressure at admission. The patients' outcome might be also influenced by healthcare system-related factors, including do-not-resuscitate (DNR) recommendation, time of admission to the hospital, and the stroke unit degree of reference. The DNR recommendation, a written instruction from a physician to all healthcare providers not to perform cardiopulmonary resuscitation, based on the clinical and radiographic predictors strongly affects the intensity of care and range of therapeutic interventions for a critically ill patient. In such cases, the phenomenon of self-fulfilling prophecy may occur, especially when the DNR recommendation is instituted within the first 24 hours since admission [11],-although regional clinical practices may vary in this field [12]. Admission on weekdays and holidays may also affect accessibility of life-saving procedures, irrespective of the patients' condition. The so-called “weekend effect” worse outcomes in patients admitted during “off-hours” have been proved in many studies concerning haemorrhagic stroke [13–15]. However, other studies did not confirm such a relationship, so the evidence seems inconsistent [16, 17]. Many authors point out that the degree of reference of the stroke center may also have an impact on mortality due to provided level of specialist interventions and care [17, 18]; therefore, they stress the importance of tertiary reference or municipal teaching centers in reducing the risk of death in the course of ICH in the last decade.

The present study investigated relationships between the day of admission (weekends and holidays vs weekdays), the clinical aspects, and the early mortality of patients with haemorrhagic stroke in tertiary multidisciplinary hospital in Poland.

## 2. Material and Methods

The retrospective analysis of the medical records was performed for all the patients diagnosed with nontraumatic intracerebral haemorrhage and admitted within 24 hours of the onset of symptoms to emergency unit of the tertiary multidisciplinary hospital between January 2008 and July 2013. The center employs 7 specialists and 6 trainees in neurosurgical department and 14 specialists and 15 trainees in neurological one. Every day (including weekends), a team consisting of one specialist and one trainee from each department is on duty. The number of nurses is reduced during off-hours (by one on each ward). Access to the CT lab, the operating room, and the anaesthesiologist/intensive care specialist service remain unchanged.

**Figure 1:**
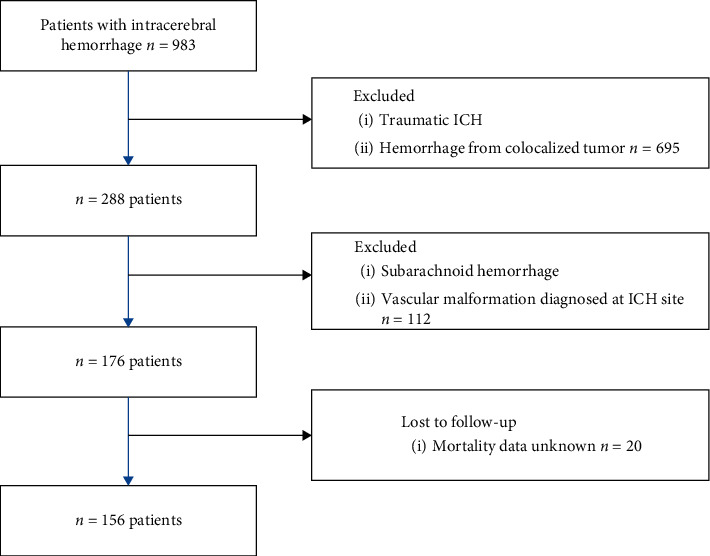
Flow Chart with inclusion and exclusion criteria.

The patients in whom haemorrhage was secondary to trauma, a ruptured aneurysm or vascular malformation, brain tumor, or primarily ischemic stroke were excluded from the studied group. Further exclusion concerned the patients who had been lost to follow-up and the data on their survival within the 30 days were not available. Finally, the studied group comprised 156 subjects (74 males, 82 females, mean age 68.7 years, SD 14, 5) (Figure 1).

The widely used Intracerebral Hemorrhage Stroke (ICH) Score [19] has been chosen to estimate the severity of stroke. The data necessary to calculate the ICH score were extracted from patients' documentation: age, sex, medical history (especially co-occurrence of hypertension, diabetes mellitus, and anaemia), level of consciousness assessed using the Glasgow Coma Scale [20], and blood pressure on admission. Other relevant information such as presence of coagulopathy, use of anticoagulants, and other comorbidities were also collected from the medical records. The radiological indices (location and volume of the haemorrhage and the presence of intraventricular bleeding) were assessed based on the CT scans performed on the day of the patients' admission. Volume was calculated according to the ABC/2 formula, where A and B are the greatest diameters of the haemorrhage perpendicular to each other and C is the approximate thickness of the stroke based on the number and thickness of CT slices [21]. All measurements were performed retrospectively by the same two trained observers, with satisfactory interrater agreement, as the error estimated by the coefficient of variation was less than 2%.

Admissions classified as “off-hours” took place at weekends (Friday after 3 pm, Saturday, and Sunday) and public holidays in Poland.

The primary outcome was defined as mortality or survival at 30 days since admission.

All the data were retrospectively analysed and concerned standard diagnostic and therapeutic procedures were carried out and documented in all the patients. The design of the study was approved by the Commission of Bioethics at Wroclaw Medical University.

## 3. Statistical Analysis

Descriptive analysis was performed for demographic and clinical characteristics for the subgroups of patients differing in 30-day outcomes as well as those admitted during weekdays or off-hours. The normality of distribution was verified with the Shapiro–Wilk test. If the normal distribution was stated, the groups were compared using the parametric *t*-student test. If the nonnormal distribution was stated, the groups were compared with the nonparametric U Mann–Whitney test and the Pearson coefficient was assessed. The ANOVA test was used to compare more than two variables in the noncombined groups. For outcome analysis, firstly, the outcome proportions among admission times were compared and then analysed in accordance with ICH and GCS scores. A score of *p* ≤ 0.05 was considered statistically significant. The statistical analysis was performed using v.10 of Statistica software.

## 4. Results

Out of the 156 patients admitted with a diagnosis of spontaneous intracranial haemorrhage, 65 died within 30 days, with an early mortality rate of 41.6%. In Table 1, the basic demographics and comorbidities of patients are presented, divided into subgroups according to the 30-day mortality outcome. They differed significantly in the presence of diabetes and hypertension: a higher incidence of those risk factors was found in the subgroup of survivors. On admission, the international normalised ratio (INR) was elevated (>2) in 10 patients (7 died within 30 days).

Out of the156 patients, 14 (9%) had their ICH evacuated in the neurosurgical department during the first 48 hours (78% of them died within 30 days), 19 patients were transferred directly to the intensive care unit (ICU) (57% died), and 127 patients were treated in the neurological department (28.3% died). Comparing the patients who had been qualified for an operation to those who had not much higher mortality was noted among the former (78% vs 32.3%; *p* < 0.001).

The mean result of the ICH scale was 1.85 SD 1.39 (ranging from 0 to 6) and was significantly higher in those who died within 30 days (2.92 vs 1.32; *p* < 0.0001). In this subgroup of patients, a statistically significant lower initial GC (8.25 vs 13.11; *p* < 0.003) score was also observed.

The ICH score was used to predict the probability of primary outcome, 30-day mortality (Table 2).

Of the 156 patients, 83 were admitted from Monday to Friday until 3 pm, and 73 were admitted during weekends or national holidays; their characteristic is shown in Table 3. There were no significant differences in treatment options between the patients admitted on weekdays or weekends (operation 14% vs. 14%; admission to ICU 14% vs. 24%, *p*=0.1). Both groups did not differ significantly in basic characteristics (mean age, sex, and cardiovascular risk factors such as hypertension and DM) and haemorrhage parameters (location, volume, and presence of intraventricular bleeding). GCS and ICH scores significantly differed at the time of admission to the hospital; the patients admitted at weekends scored higher in both scales (12.1 vs. 10.4, *p* < 0.05 and 1.5 vs. 0.53, *p* < 0.001, respectively).

On nonparametric analysis of the relationship between 30-day mortality and hospital admission time, the groups did not differ significantly (*χ*2 = 2.21, *p*=0.14). The risk of a poor outcome was three times higher for those admitted on Saturdays (OR 3.38, 95% CI 1.2–9.48, *p* < 0.05).

Adjusting this basic model to the severity of haemorrhage using the ICH scale, a significant effect of the admission time was observed for patients with an ICH score of 1 (14 patients admitted on the weekend, 26 patients on weekdays) and 3 (15 and 12 patients, respectively) (Table 4). Adjusting the model to the GCS scale, no statistical differences were noted for patients admitted on weekdays vs. weekends and holidays (Table 5).

Further analysis of early patient mortality regarding all mentioned variables (date of admission, ICH, and GCS) was not performed due to the small number of patients in subgroups.

## 5. Discussion

We aimed to analyse the relationships between early mortality risk and factors associated strictly with hospital care. The DNR recommendation, which is relatively rarely established in Polish conditions, was not applied in any of the patients included in the study [11]. We chose the stroke unit in the university center, with the highest degree of reference in the region, to minimise the possibility of deficiency in the full spectrum of specialist intervention and care, which may occur in rural healthcare facilities. Although some authors postulated an impact of the educational cycle schedule in teaching centers on stroke patients' mortality, most of the published data do not support this observation [22, 23].

Ultimately, we focused on a potential link between the day of admission and early mortality in haemorrhagic stroke. In the studied group, no increased risk of unfavourable outcome was observed for the patients admitted during weekends vs weekdays. The findings suggest that the quality of standard procedures and treatment options in this particular medical setting did not depend on the day of admission. The “weekend adverse effect” has been demonstrated in many studies on haemorrhagic stroke [13–15], although standardised care provided by stroke units was postulated to reduce this [16, 17, 23, 24]. A similar percentage of the patients operated and admitted to the intensive care unit irrespective of admission time also contradicts the “weekend effect” in our study. The higher mortality among operated patients (78%) in comparison with others (32%) most probably resulted from a more severe neurological condition and thus unfavourable prognosis in the former (GCS 8.3 vs. 11.6; *p* < 0.005). Patients admitted to the intensive care unit had worse baseline ICH than those handled in the neurological department and presented with more comorbidities and developed early respiratory insufficiency, which explains the higher mortality in this subgroup.

However, the etiology and case severity of ICH should be taken into account while considering the relationships between the time of admission and mortality in haemorrrhagic strokes, which has not been consistently done in the mentioned studies [13–17, 23–25]. For more relevant findings, we aimed to investigate a group of patients with possibly homogenous ICH etiology. According to the SMASH-U classification [25], cases with trauma, tumor or cerebrovascular malformations, preceding ischemic stroke, or subarachnoid haemorrhage were excluded from the studied group, especially as most of the listed conditions are associated with relatively high in-hospital mortality [25]. Many authors [16, 18, 26] reported a more severe condition (measured mostly by GCS) in the patients with haemorrhagic stroke admitted at weekends, which is consistent with findings of this study (patients admitted during weekends or holidays had lower GCS and higher ICH scores).

Good prognostic accuracy of the ICH scale alone (with regard to early mortality) was demonstrated in the studied group; similar to the outcomes reported by authors of this scale, only patients who scored 4 on the ICH scale had a higher risk of mortality than that reported in the literature [19, 25, 27, 28]. Also, patients with ICH score 1 or 3 admitted during off-hours (17% of all analysed groups) had a higher risk of death. This result suggests the influence on the prognosis of not only out-of-hospital factors that cause patients to reach a hospital in more severe conditions at weekends. Some researchers have paradoxically found the “reverse off-hour effect”: the median time from onset of ICH symptoms to admission, neuroimaging, and therapeutic decision was shorter for patients admitted outside of normal working hours [16, 29]. However, patients' more severe condition results in a shorter time in emergency transport to a hospital and a shorter time to undertake inpatient medical interventions [26]. Thus, patients in a relatively good initial condition (ICH score 1 or 3) may not necessarily benefit from immediate prehospital intervention, especially during off-hours. Moreover, patients within the same range of GCS score (a clinical component of the ICH scale) have similar risk of death whether admitted on weekends or weekdays. The findings in this field are inconsistent since some authors who have taken into account the severity of stroke have reported a positive relationship between admission time and outcome [29, 30], while others have not [16, 24, 31].

It is also worth considering that patients' severe condition on admission might result from the delay in access to the emergency unit. Various factors may determine recognition of the disease onset and transfer to a hospital. Disturbed consciousness, syncope or seizures, greater disability at stroke onset, and previous myocardial infarction, as well as emergency ambulance transport, were found to correlate with shorter admission times to hospital [32]. Minor symptoms with nocturnal onset and living alone were not reported to influence admission time to hospital [26, 32], while symptoms related to the posterior circulation area (impaired vision, instability) and headache [32], older age, and limited access to medical services were associated with its significant delay [21, 26]. We assumed the time range of 24 hours between the onset of symptoms and admission to the emergency unit, but the specific time to admission was not estimated; this additional factor deserves attention in further studies in this field.

Our study presents a systematic analysis of clinical and healthcare-related factors affecting patients' early mortality with ICH, admitted to the tertiary stroke center. The study's limitations include the relatively small sample size, which made it challenging to perform the multivariate analysis of all considered issues. The retrospective type of study could also be perceived as its limitation. Still, we believe that the findings reflect clinical practice experience and may be relevant for improving the effectiveness of the care for such patients. Further prospective observation, potentially including other stroke units, might be undertaken to provide a better insight into the factors determining outcome in ICH.

## 6. Conclusions

Patients with ICH admitted to the specialist stroke center during the off-hours present with more severe clinical condition. However, for those with milder neurological deficit, the admission during weekends is associated with higher risk of mortality. Clinical issues determining the severity of the patients' condition remain the most significant prognostic factors for mortality. To lower the mortality rate among patients with hemorrhagic stroke, it seems necessary to standardize round-the-clock emergency services at the prehospital and inpatient stage.

## Figures and Tables

**Table 1:** Univariate analysis of patient characteristics.

	All (*n* = 156)	Survival (*n* = 91)	Death (*n* = 65)	*p* value
Mean age	68.72 (SD 14.5)	67.02 (SD 13.7)	71.1 (SD 15.3)	0.08
Age >80 years	46 (29.5%)	21 (23%)	25 (38.5%)	0.02

Sex				
Male	74	45 (60.8%)	29 (39.1%)	
Female	82	46 (56.1%)	36 (43.9%)	0.55

Hypertension	114 (73.1%)	73 (80.2%)	41 (63.1%)	0.02
DM	20 (12.9%)	17 (18.7%)	3 (4.6%)	0.01

GCS score				
13–15	84 (53.8%)	64 (70.3%)	20 (30.8%)	<0.001
5–12	60 (38.5%)	26 (28%)	34 (52.3%)	<0.003
3-4	12 (7.7%)	1 (1.1%)	11 (16.9%)	<0.001

Location of ICH				
Infratentorial	25 (16%)	12 (13%)	13 (20%)	
Supratentorial	131 (83%)	79 (86,8%)	52 (80%)	0.25

ICH volume				
>30 ml	69 (44.2%)	7 (29.7%)	42 (64.6%)	
<30 ml	87 (55.7%)	64 (70.3%)	23 (35.4%)	<0.001

Intraventricular haemorrhage	64 (41%)	25 (27.5%)	39 (60%)	<0.001

**Table 2:** Prediction of 30-day mortality death using the ICH scale alone.

ICH scale	*n*	OR	95% CI	*p* value
0	29	0.11	0.02–0.49	0.004
1	40	0.21	0.08–0.62	0.003
2	40	0.83	0.37–1.86	0.65
3	27	3.5	1.39–9	0.007
4	13	7.2*∗*10^11^	n/a	0.99
5	6	10	1.1–89.9	0.04
6	1	n/a	n/a	n/a

n/a, not applicable.

**Table 3:** Basic analysis of patient characteristics admitted on weekdays and off-hours.

	Weekdays (*n* = 83)	Weekends (*n* = 73)	*p* value
Mean age	68.23 (SD ± 15.2)	69.29 (SD ± 13.7)	0.65
Age >80 years	27 (32.5%)	20 (27.4%)	0.48

Sex			
Male	44 (53%)	43 (59%)	
Female	39 (47%)	0 (41.1%)	0.45

Hypertension	64 (77.1%)	51 (69.9%)	0.3
DM	9 (10.8%)	12 (16.4%)	0.31

GCS score			
13–15	51 (61.4%)	33 (45.2%)	
5–12	28 (33.7%)	32 (43.8%)	
3-4	4 (4.8%)	8 (10.9%)	0.03

Location of ICH			
Infratentorial	11 (13.2%)	14 (19.2%)	
Supratentorial	72 (86.7%)	59 (80.8%)	0.31

ICH volume			
>30 ml	35 (42.2%)	34 (46.6%)	
<30 ml	48 (57.8%)	39 (53.4%)	0.58

Intraventricular haemorrhage	33 (39.7%)	31 (42.5%)	0.73
30-day mortality	35 (47.9%)	30 (36.1%)	0.18

**Table 4:** Risk of 30-day mortality based on the Intracerebral Haemorrhagic Stroke Score regarding admission day (weekdays vs. weekends and holidays).

	OR	95% CI	*p*
ICH 0	0.38	0.08–1.86	0.23
ICH 1	0.09	0.01–0.76	0.03
ICH 2	0.8	0.26–2.49	0.71
ICH 3	7.24	1.4–36.9	0.02
ICH 4	2.3*∗*10^11^	n/a	0.23
ICH 5	7.8	0.8–73	0.07

**Table 5:** Risk of 30-day mortality based on the Glasgow Coma Scale score with regard to the day of admission versus GCS 13–15 (weekdays vs off-hours).

	OR	95% CI	*p*
GCS 5–12	1.44	0.01–3.75	0.44
GCS 3-4	0.35	0.01–1.11	0.07

## Data Availability

The numerical data used to support the findings of this study are available from the first (marnow64@interia.pl) and corresponding authors (marta.waliszewska@gmail.com) upon request.
